# ﻿A new cryptic species of terrestrial breeding frog of the *Pristimantisdanae* Group (Anura, Strabomantidae) from montane forests in Ayacucho, Peru

**DOI:** 10.3897/zookeys.1187.104536

**Published:** 2023-12-20

**Authors:** Valia Herrera-Alva, Alessandro Catenazzi, César Aguilar-Puntriano

**Affiliations:** 1 Departamento de Herpetología, Museo de Historia Natural de la Universidad Nacional Mayor de San Marcos, Lima, Peru Museo de Historia Natural de la Universidad Nacional Mayor de San Marcos Lima Peru; 2 Laboratorio de Sistemática y Ecología de Vertebrados, Facultad de Ciencias Biológicas, Universidad Nacional Mayor de San Marcos, Lima, Peru Universidad Nacional Mayor de San Marcos Lima Peru; 3 Florida International University, Department of Biological Sciences, 11200 SW 8th Street, Miami, FL 33199, USA Florida International University Miami United States of America

**Keywords:** Chytridiomycosis, cryptic species, montane forests, morphology, phylogeny, Bosques montanos, especies crípticas, filogenia, morfología, quitridiomicosis

## Abstract

Based on morphological and molecular characters, we describe a new species of terrestrial breeding frog of the *Pristimantisdanae* Group from montane forests of La Mar Province, Ayacucho Department in southern Peru, at elevations from 1200 to 2000 m a.s.l. The phylogenetic analysis, based on concatenated sequences of gene fragments of 16S rRNA, RAG1, COI and TYR suggests that the new species is a sister taxon of a clade that includes one undescribed species of *Pristimantis* from Cusco, *Pristimantispharangobates* and *Pristimantisrhabdolaemus*. The new species is most similar to *P.rhabdolaemus*, which differs by lacking scapular tubercules and by its smaller size (17.0–18.6 mm in males [n = 5], 20.8–25.2 mm in females [n = 5] in the new species vs. 22.8–26.3 mm in males [n = 19], 26.0–31.9 mm in females [n = 30] of *P.rhabdolaemus*). Additionally, we report the prevalence of *Batrachochytriumdendrobatidis* (Bd) in this species.

## Abstract (es)

Describimos una nueva especie de rana terrestre de desarrollo directo del grupo *Pristimantisdanae* de bosques montanos procedentes de la provincia de La Mar, departamento de Ayacucho al sur de Perú con rango de distribución altitudinal entre los 1200–2000 msnm, en base a caracteres morfológicos y moleculares. El análisis filogenético basado en las secuencias concatenadas de los fragmentos de genes ARNr 16S, COI, RAG1 y TYR sugiere que la nueva especie es un taxón hermano del clado que incluye a una especie de *Pristimantis* no descrita de Cusco, *Pristimantispharangobates* y *Pristimantisrhabdolaemus*. La nueva especie se asemeja más a *P.rhabdolaemus*; de la cual difiere por la ausencia de tubérculos escapulares y su menor tamaño corporal (17.0–18.6 mm en machos [n=5], 20.8–25.2 mm en hembras [n=5] en la nueva especie vs 22.8–26.3 mm en machos [n=19], 26.0–31.9 mm en hembras [n=30] de *P.rhabdolaemus*). Adicionalmente, reportamos la prevalencia de *Batrachochytriumdendrobatidis* (Bd) en esta especie de Terrarana.

## ﻿Introduction

*Pristimantis* (Terrarana, Strabomantidae) is an amphibian genus that comprises more than 600 species and occurs thoughout the Americas (Hedges et al. 2008; Padial and De La Riva 2009; Padial et al. 2014; Waddell et al. 2018) from Honduras to Argentina. In Peru, there are 145 *Pristimantis*, which represents more than 20% of its global richness (Frost 2023). Many species of *Pristimantis* are morphologically similar despite belonging to different lineages (Elmer and Cannatella 2008; Padial and De la Riva 2009; Siqueira et al. 2009; Kieswetter and Schneider 2013; Hutter and Guayasamin 2015; Ortega-Andrade et al. 2015). The ubiquity of cryptic species in *Pristimantis* has led to underestimation of the real number of species in the genus (Ortega-Andrade et al. 2015; Guayasamin et al. 2017; Paez and Ron 2019; Carrion-Olmedo and Ron 2021). Nevertheless, the application of molecular techniques in an integrative framework (Dayrat 2005) generated a steady increase in species richness of *Pristimantis* (Köhler et al. 2022; Reyes-Puig and Mancero 2022). Integrative taxonomy uses more than one line of evidence and discipline to infer species limits (Simpson 1961; Wiley 1978; De Queiroz 2005; Schlick-Steiner et al. 2010; Aguilar et al. 2013; Hutter and Guayasamin 2015) and has become a critical tool to identify and delimit species as well as in understanding species formation (Aguilar et al. 2013; Minoli et al 2014; Rojas et al. 2018; Hillis 2019; Zozaya et al. 2019).

One group with cryptic species includes *Pristimantisrhabdolaemus*. The taxonomic history is complex because Lynch and McDiarmid (1987) synonymised *Pristimantispharangobates* with *P.rhabdolaemus*, until Lehr (2007) again split these two cryptic species. Incorrect labelling of GenBank sequence EF493706 (uploaded during the period from 1987 to 2007 when synonymy was in place) of *P.pharangobates* as “*P.rhabdolaemus*” (Heinicke et al. 2007; Padial et al. 2014; Lehr et al. 2017a, b; Acevedo et al. 2020) contributed to taxonomic confusion. Furthermore, specimens from Bolivia incorrectly assigned to *P.rhabdolaemus* added more confusion. Despite this history, *P.rhabdolaemus* species limits have not been studied using integrative taxonomy.

Therefore, as part of a study of *Pristimantisrhabdolaemus* species boundaries, we collected direct development frogs from the montane forests of La Mar Province, Ayacucho Department. Molecular and morphological analyses revealed that the collected specimens belong to an undescribed species. Our goals are to describe the new species and infer its relationships with other species of the *Pristimantisdanae* species Group, as well as provide information about infection by the fungus *Batrachochytriumdendrobatidis* (Bd).

## ﻿Materials and methods

### ﻿Fieldwork and voucher specimens

**Figure 1.**
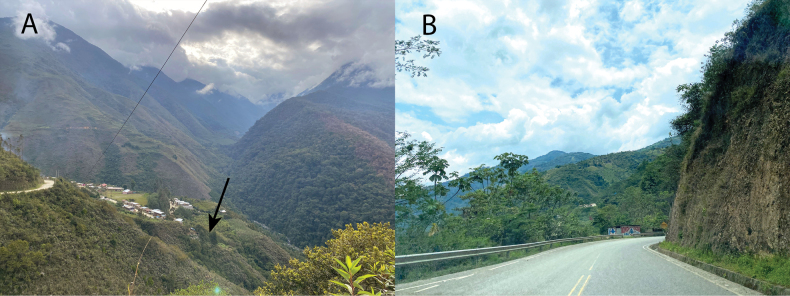
Montane forest of Cajadela (**A**) and Machente (**B**), Ayacucho Department. Type locality of *Pristimantissimilaris* sp. nov. in Cajadela (**A**). Note the presence of secondary forest in both localities. Photo **A** taken on 8 November 2021 and **B**, on 24 October 2022. Arrow marks the type locality.

VHA conducted field research in the montane forest of two small valleys (Fig. 1) in the VRAEM (Spanish acronym for Valley of the Rivers Apurimac, Ene and Mantaro), Ayacucho Department, southern Peru. The fieldwork was organised in two stages. The first occurred from November 2018 to June 2019 in the valley of the Chunchubamba River, which goes from Chiquintirca (2900 m a.s.l.) to San Antonio (800 m a.s.l.) in the districts of Anco and Anchihuay (both from La Mar Province). The second field-trip was in November 2021 in the valley of the Piene River, which goes from Yanamonte (2900 m a.s.l.) to San Francisco (800 m a.s.l.) in the Districts of Sivia (Huanta Province) and Ayna (La Mar Province), which included the visit to the type locality of *P.rhabdolaemus* in Machente (1650 m a.s.l), also previously known as Huanhuachayocc, a name no longer recognised by the locals.

﻿We extracted liver tissues by pulling the liver out of a small abdominal incision. We fixed specimens in 10% formaldehyde and stored them in 70% ethanol in the Department of Herpetology of the Museo de Historia Natural Universidad Nacional Mayor de San Marcos (MUSM), Lima, Perú. The Ministry of Agriculture issued research, collecting and genetic resources permits (000063-2018-GRA/GG-GRDE-DRAA-DFFS-D, 029-2016-SERFOR-DGGSPFFS and D000012-2022-MIDAGRI-SERFOR-DGGSPFFS-DGSPFS).

### ﻿Morphology and morphometry

We follow Lynch and Duellman (1997) for the description format, except that we use “vomerine odontophores” instead of “dentigerous processes of vomers” (Duellman et al. 2006) and Duellman and Lehr (2009) for diagnostic characters. We sexed specimens by examining for the presence of vocal slits in mature males and by inspecting gonads in dissected specimens. VHA measured the following distances to the nearest 0.1 mm with digital calipers under a stereomicroscope: 1) snout-vent length (**SVL**), 2) tibia length (**TL**, ﻿distance from the knee to the distal end of the tibia), 3) foot length (**FL**, distance from the proximal margin of inner metatarsal tubercle to tip of Toe IV), 4) head length (**HL**, from the angle of the jaw to tip of snout), 5) head width (**HW**, at the level of the angle of the jaw), 6) horizontal eye diameter (**ED**), 7) horizontal tympanum diameter (**TY**), 8) interorbital distance (**IOD**), 9) upper eyelid width (**EW**), 10) internarial distance (**IND**), 11) eye-nostril distance (**EN**), straight line distance between anterior corner of orbit and posterior margin of external nares) and one extra measurement: 12) Finger IV disc width (**F4**). ﻿Fingers and toes are numbered pre-axially to postaxial from I–IV and I–V, respectively. We compared the lengths of toes III and V by adpressing both toes against Toe IV; lengths of fingers I and II were compared by adpressing the fingers against each other. Vladimir Díaz Vargas photographed live specimens and VHA preserved the specimens. We used photographs for descriptions of skin texture and colouration. ﻿Specimens examined are listed in Appendix 1; other collection acronyms are MUSM = Museo de Historia Natural San Marcos (Lima, Peru); KU = University of Kansas, Museum of Natural History (Kansas, USA); LSUMZ = Louisiana State University Museum of Zoology (Louisiana, USA).

### ﻿Molecular phylogenetic analysis

We performed a phylogenetic analysis to infer relationships of the new species with other species of the *Pristimantisdanae* Group (Padial et al. 2014). We used fragments of 16S rRNA, COI, RAG1 and TYR genes. We obtained novel sequences by extracting DNA from six specimens of the new species (MUSM 41030, 41031, 41035, 41036, 41037, 41323) with a commercial extraction kit (IBI Scientific, Iowa, USA). We followed Hedges et al. (2008) and von May et al. (2017) for primers and PCR thermocycling conditions. Primers are listed in Table 1. We purified PCR products using Exosap-IT Express (Affymetrix, Santa Clara, CA, USA). MCLAB (San Francisco, CA) performed Sanger sequencing.

**Table 1.** Primers employed in this study for PCR and DNA sequencing. F = forward, R = reverse.

Locus	Primer		Sequence (5’ – 3’)	Reference
**16S**	16SAR	F	CGCCTGTTTATCAAAAACAT	Meyer (2003)
	16SBR	R	CCGGTCTGAACTCAGATCACGT	
**COI**	dgLCO1490	F	GGTCAACAAATCATAAAGAYATYGG	Bossuyt and Milinkovitch (2000)
	dgHCO2198	R	TAAACTTCAGGGTGACCAAARAAYCA	
**RAG1**	R182	F	GCCATAACTGCTGGAGCATYAT	Heinicke et al. (2007)
	R270	R	AGYAGATGTTGCCTGGGTCTTC	
**TYR**	Tyr1C	F	GGCAGAGGAWCRTGCCAAGATGT	Lanfear et al. (2012)
	Tyr1G	R	TGCTGGGCRTCTCTCCARTCCCA	

We follow Padial et al. (2014) and Pyron and Wiens (2011) for species group assignment within *Pristimantis* and the choice of outgroup taxa. We downloaded from GenBank all available sequences of 16S rRNA, COI, RAG1 and TYR of other species of the *P.danae* Group and some of the outgroup taxa. We used selected species of the *P.conspicillatus* Group (*P.bipunctatus*, *P.iiap* and *P.skydmainos*) and *P.prolatus* as outgroup taxa (Appendix 2).

We used Geneious Prime version 2023.0.1 (Biomatters, http://www.geneious.com/) to assemble pair-end reads, generate a consensus sequence for each gene and align our novel and GenBank sequences with MAFFT included in Geneious as a plug-in. Posteriorly, we concatenated the four genes (16S, COI, RAG1 and TYR) using the default settings in Geneious. We trimmed aligned sequences to a length of 591 bp for 16S, 685 bp for COI, 624 bp for RAG1 and 545 bp for TYR (Fasta file included in doi: 10.5281/zenodo.8278333). To obtain the nucleotide substitution model for each gene, we used PartitionFinder with Python v. 2.7 + Anaconda2 (Lanfear et al. 2017). We inferred a Maximum Likelihood phylogenetic tree withIqTree ﻿(Nguyen et al. 2015) by using its web server (http://iqtree.cibiv.univie.ac.at/) with the following settings: ultrafast bootstrap method, 1000 bootstrap alignments and nucleotide evolution models of GTR+I+G for the gene 16S and for 1^st^ codon position of COI; GTR+G for RAG1, TYR and 3^rd^ codon position of COI; and GTR for 2^nd^ codon positions of COI. Additionally, we generated a tree using Bayesian Inference using the plug-in MrBayes for Geneious Prime with 1.1 × 10^6^ generations and sampling every 200 generations from the Markov Chain Monte Carlo (MCMC). We determined stationarity by plotting the log-likelihood scores of sample points against generation time; when the values reached a stable equilibrium and split frequencies fell below 0.01, stationarity was assumed. We discarded 100,000 samples and 10% of the trees as burn-in. We consider Bayesian Posterior Probabilities (BPP) > 95% as evidence of support for a clade (Huelsenbeck and Ronquist 2001; Wilcox et al. 2002; Aguilar et al. 2013). We visualised both trees in FigTree v.1.4.4.

**Figure 2.**
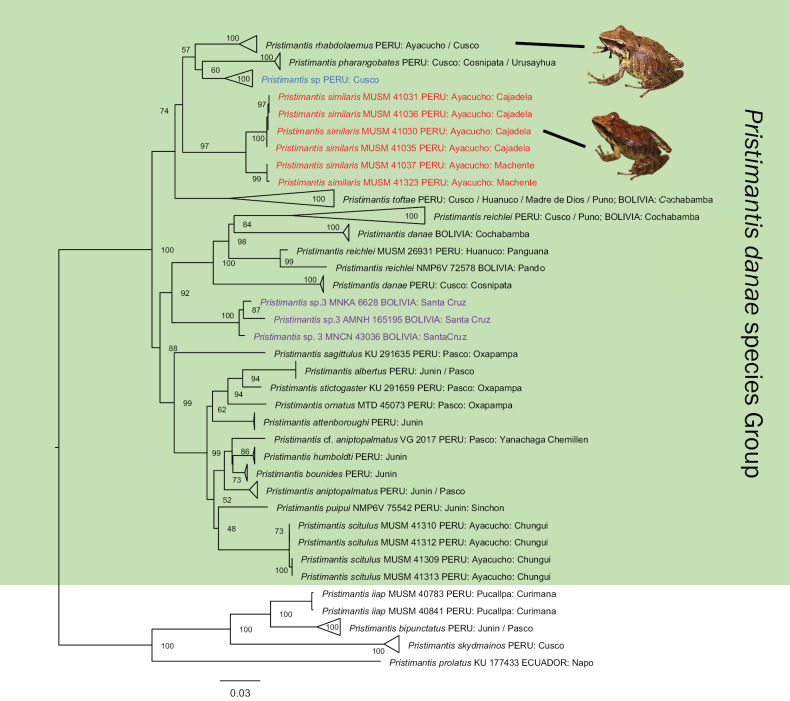
Maximum Likelihood tree of concatenated genes 16S rRNA, COI, RAG1 and TYR taken from GenBank and novel sequences. Numbers on nodes are bootstrap values (see Materials and Methods section for details). Green shadow corresponds to the ingroup. *Pristimantissimilaris* sp. nov. in red, *Pristimantis* sp. 3 from Bolivia in purple and *Pristimantis* sp. from Cusco in blue. Maximum Likelihood optimal tree with bootstrap node values from the analysis of a concatenated dataset of 591 bp (16S), 685 bp (COI), 624 bp (RAG1) and 545 bp (TYR) of 128 species aligned by MAFFT and node support assessed using 10,000 replicates in IQTREE.

Finally, we compare uncorrected p-distances of 591 bp (including gaps) of 16S mithocondrial rRNA gene of *Pristimantis* included in our analysis (included as a separated file in: doi: 10.5281/zenodo.8278333). To estimate p-uncorrected genetic distances, we used MEGA 11 (Tamura et al. 2021) with a variance estimation method of 1000 bootstrap and rates amongst sites of G+I. We excluded from this analysis species from the sister clade (*P.bounides*, *P.aniptopalmatus*, *P.albertus*, *P.attenboroughi*, *P.humboldti*, *P.danae*, *P.ornatus*, *P.puipui*, *P.reichlei* and *P.sagittulus*, Fig. 2), except from *Pristimantis* sp.3 from Bolivia because these specimens had been identified as *P.rhabdolaemus* on GenBank and *P.scitulus*, because they are novel sequences for this species.

### ﻿Quantitative PCR assays for fungal infection (Bd)

During fieldwork in 2018, 2019 and 2021, we swabbed live frogs of the new species with a synthetic dry swab (Medical Wire & Equipment #113) to quantify infection by *Batrachochytriumdendrobatidis* (Bd). We stroked swabs across the skin of juveniles and adults a total of 30 times per individual: five strokes on each side of the abdominal mid-line, five strokes on the inner thighs of each hind leg and five strokes on the foot webbing of each hind leg. We used a standard quantitative Polymerase Chain Reaction (qPCR) assay using DNA extracted from swabs to quantify the level of infection (Boyle et al. 2004). Following the protocol of Boyle et al. (2004) and Hyatt et al. (2007), we extracted DNA from swabs using 40 µl of PrepMan Ultra (Applied Biosystems). We analysed each extract once with a QuantStudio 3 qPCR system (ThermoFisher Scientific). We calculated the number of zoospore equivalents (ZE) (i.e. the genomic equivalent for Bd zoospores) by comparing the sample results with a serial dilution of standards (gBlock synthetic standards, IDT DNA, Iowa, USA). We considered any sample with ZE > 1 to be infected or Bd-positive.

### ﻿Nomenclatural acts

The electronic version of this article in Portable Document Format (PDF) will represent a published work according to the International Commission on Zoological Nomenclature (ICZ) and, hence, the new name contained in the electronic version is effectively published under that Code from the electronic edition alone. This published work and its nomenclatural acts have been registered in ZooBank, the online registration system for the ICZN. The ZooBank LSIDs (Life Science Identifiers) can be resolved and the associated information is viewed through any standard web browser by appending the LSID to the prefix http://zoobank.org/. The LSID for this publication is urn: urn:lsid:zoobank.org:pub:226A24AB-B4BE-4EFD-BF11-D6CA719AB601.

**Figure 3.**
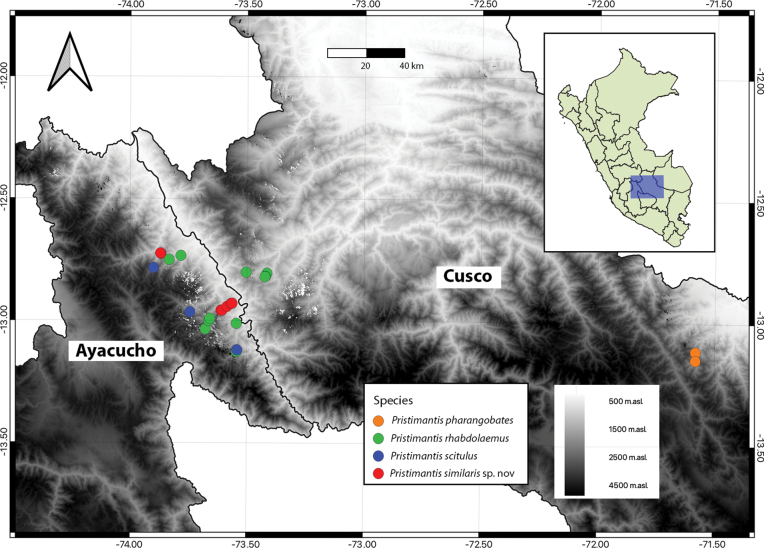
Distribution map of some species of the *P.danae* species Group in Ayacucho and Cusco Departments. Raster of altitude from 500 to 4500 m. a.s.l. (white to black). Locality of new species in red circles.

## ﻿Results

### ﻿Molecular phylogenetic analysis

Our Maximum Likelihood phylogeny, based on four concatenated gene fragments (Fig. 2 and Appendix 3 – expanded ML tree), found the new species (in red) included in a clade with specimens of *Pristimantisrhabdolaemus*, *P.pharangobates* and *P.toftae* from their respective type localities, as well as a candidate species from Cusco (in blue). The results from the Bayesian phylogeny (Appendix 4) are largely congruent with the results from the ML phylogeny.

*Pristimantisscitulus* is within the *danae* Group and sister to a clade consisting of *P.aniptopalmatus*, *P.humboldti* and *P.bounides*, but with low support. Both our ML and BI phylogenies recover *P.danae* as paraphyletic, with individuals from the type locality in Cosñipata (Cusco, Peru) forming part of a clade that includes *P.danae* specimens from Bolivia and *P.reichlei*, albeit with low support.

﻿Genetic distances (uncorrected p-distances) for the rRNA 16S gene between *P.similaris* sp. nov. and species of the *P.danae* species Group vary from 5.6–6.9% for *P.rhabdolaemus*, 5.9–6.3% for *P.pharangobates*, 6.1–6.7% for *Pristimantis* sp., 6.5–7.5% for *P.scitulus*, 7.3–7.9% for *Pristimantis* sp. 3 and 7.7–9.3% for *P.toftae* (see Suppl. material 1, doi: 10.5281/zenodo.8278333). We also identified two populations within our new species, the first one from the type locality in Cajadela and the second, from Machente. The genetic distances between these populations were 2.7–2.8%.

### ﻿Fungal infection by *Batrachochytriumdendrobatidis* (Bd)

We found six out of 18 specimens of *P.similaris* (30%) infected by the fungus *Batrachochytriumdendrobatidis* (Bd), with levels of infection varying from 11.5 to 8889.3 zoospore genomic equivalents. Our finding confirms that species of *Pristimantis* can be infected with the fungus (Catenazzi et al. 2011; Warne et al. 2016), despite their life cycle excluding aquatic stages and, thus, limiting the frogs’ exposure to the aquatic zoospores of Bd.

### ﻿Species description

Our phylogenetic tree shows a candidate species from Ayacucho with high support and having high genetic distances with closely-related phylogenetic species (see Fig. 2 and Appendix 3). In addition, after a careful examination of its morphology and pattern of colouration, this candidate species shows differences with other species of the *P.danae* Group. Based on these results, we describe this candidate species from Ayacucho Department as a new species of *Pristimantis*.

#### ﻿ Pristimantis similaris sp. nov.

Taxon classificationAnimaliaAnuraStrabomantidae

106D7E7B-730C-5CAC-B3E9-22BD2BD5E452

https://zoobank.org/BC56FD8A-6EBD-43C9-A446-689FC3253576

Figs 4 , 5 , 6

##### Common name.

﻿English: Similar Rubber Frog. Spanish: Rana cutín similar.

##### Generic placement.

**Figure 4.**
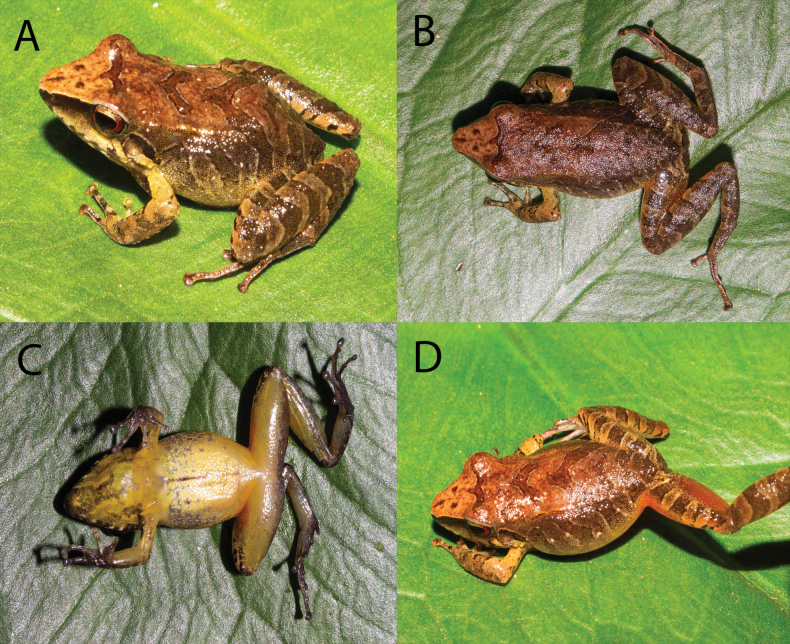
*Pristimantissimilaris* sp. nov. (**A–D**) male. SVL: 17.0 mm. Holotype. MUSM 41030. Photos by Vladimir Diaz-Vargas.

﻿We assign this species to the genus *Pristimantis*, based on morphology and molecular data (Figs 2, 4, 6).

##### Type material.

**Figure 5.**
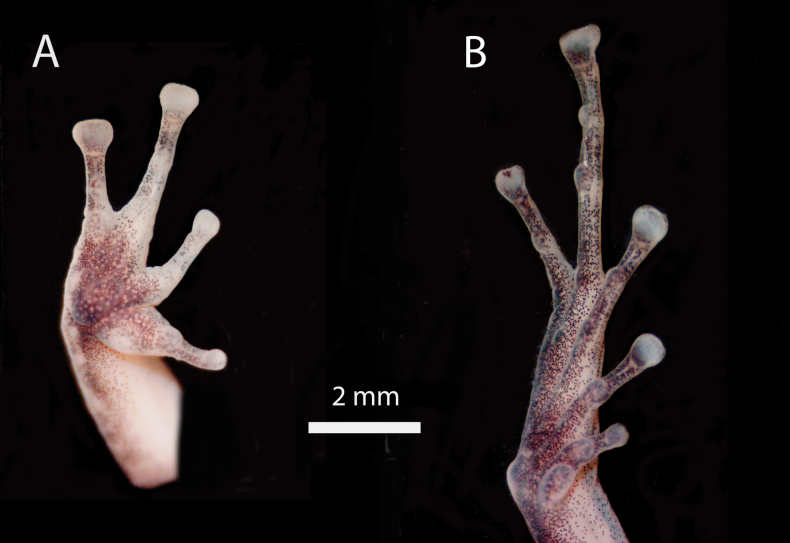
*Pristimantissimilaris* sp. nov. **A** hand **B** toe. Male. Holotype. MUSM 41030. Photos by VHA.

***Holotype*.**MUSM 41030, adult male (Figs 4, 5) from Comunidad Cajadela (12°57'16.50"S, 73°35'0.70”’W, 1460 m a.s.l.), Distrito Anco, Provincia La Mar, Departamento Ayacucho, Peru, collected on 15 November 2018 by V. Herrera-Alva, E. Castillo-Urbina, V. Díaz, M. Fernandez, and J. Gamboa.

***Paratypes*.** Nine specimens. Five adult females (MUSM 41031, 41032, 41035, 41036 and MUSM 41037). Four adult males (MUSM 41029, 41028, 41033 and MUSM 41034). All the specimens were collected at the type locality, except MUSM 41037, which was collected in Comunidad Machente (12°41'31.70"S, 73°51'0.30"W, 1640 m a.s.l.), Distrito Ayna, Provincia La Mar, Departamento Ayacucho, Peru, on 11 November 2021 by V. Herrera-Alva, E. Castillo-Urbina, V. Díaz and K. Ñaccha.

##### Diagnosis.

**Figure 6.**
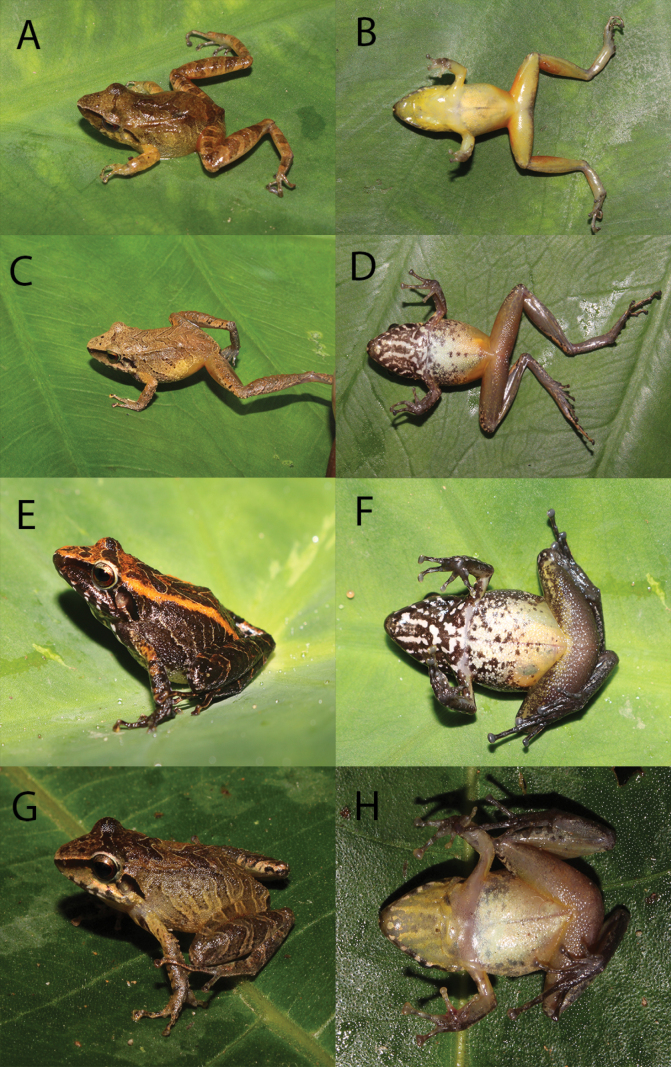
**A–H** colour and pattern variation of *Pristimantissimilaris* sp. nov. Specimen from **A–F** collected in Cajadela: **A, B** male MUSM 41029 **C, D** female MUSM 41031 **E, F** female MUSM 41032. Specimen **G, H** male MUSM 41326 collected in Machente. Photos by V. Diaz-Vargas and E. Castillo-Urbina.

﻿A new species of *Pristimantis* assigned to the *P.danae* species Group having the following combination of characters: (1) Skin on dorsum shagreen, skin on venter areolate; discoidal and dorsolateral folds present, weak; thoracic fold present; (2) tympanic membrane and tympanic annulus present, distinct, visible externally; (3) snout subaccuminated in dorsal view, round in lateral view; (4) upper eyelid lacking tubercles; EW smaller than IOD; cranial crest absent; two small and flat tubercles above the snout near the eyes; (5) dentigerous processes of vomers low, oblique in five of the paratypes, absent in four paratypes and the holotype; (6) males with vocal slits, subgular vocal sac large extending on to chest and without nuptial pads; (7) Finger I slightly shorter than Finger II; discs of digits expanded, flat and truncated; (8) fingers without lateral fringes; (9) ulnar tubercles present, but diffuse; (10) heel with two to three small and flat tubercles; inner tarsal fold present, small; (11) inner metatarsal tubercle ovoid, 2–3 times larger than outer one; outer metatarsal tubercle small, ovoid; ﻿numerous and flat supernumerary tubercles; (12) toes without lateral fringes; basal toe webbing absent; toe V is slightly longer than toe III; toe discs about as large as those on fingers; (13) in life, dorsum varies from blackish to dark brown with three conspicuous chevrons (not very visible in some cases) (Fig. 6); in most of the adults, the anterior surfaces of thighs reddish-orange, posterior surfaces of thighs brown; flanks cream without tubercles; groin same pattern as flanks mostly, some specimens with orange-reddish colouration (Figs 4, 6); venter cream to yellow with black conspicuous reticulations in the throat and black marks in the chest, males present yellow throat with black or white longitudinal reticulations not as conspicuous as in females (Figs 4, 6); iris dark copper-coloured with fine black vermiculations; (14) SVL in adult females 20.8–25.2 mm (mean = 23.4 ± 1.8 SE, n = 5), in adult males 17.0–18.6 mm (mean = 18.1 mm ± 0.7 SE, n = 5).

##### Comparisons.

*Pristimantissimilaris* ﻿is distinguished from its congeners in Peru and Bolivia by the following combination of characters: skin on dorsum areolate, tympanum and tympanic annulus distinct, weakly-defined discoidal and dorsolateral folds, two small and flat tubercles above the snout near the eyes (not conspicuous in preservative), dorsum dark brown with three darker chevrons, anterior surface of thighs usually orange-reddish and posterior surface of thighs dark brown. *Pristimantissimilaris* can be distinguished from *P.rhabdolaemus* and *P.pharangobates* by the following characters (characters in parenthesis): smaller SVL of 20.8–25.8 mm in ten females and 15.2–18.9 mm in eight males (*P.pharangobates* 23.1–27.8 mm in females and 15.2–18.2 mm in males; *P.rhabdolaemus* 25.5–31.9 mm in females and 24.1–26.3 mm in males); absence of scapular tubercles (present in both species); presence of conspicuous longitudinal black and white or yellow marks on the throat and chest (less conspicuous in *P.pharangobates* and *P.rhabdolaemus*).

Other species in the *Pristimantisdanae* species Group that are similar to *P.similaris* include *P.danae*, *P.reichlei*, *P.scitulus* and *P.toftae*. *Pristimantisdanae* and *Pristimantisreichlei* also have brown chevrons in the dorsum and differ from the new species by the combination of the following characters: males nuptial pads absent (present in *P.danae* and *P.reichlei*), dorsolateral folds present (weak in *P.danae* and absent *P.reichlei*), small pale spots in the posterior surfaces of the thighs absent (present in *P.danae* and *P.reichlei*) and smaller size in *P.similaris*. *Pristimantisscitulus* is morphologically similar to *P.similaris* and has a parapatric distribution (Yuraccyacu, in the Piene Valley, Ayacucho Region). *Pristimantissimilaris* can be distinguished by lacking a conical tubercle in the upper eyelid and heels (present in *P.scitulus*), mid-ventral line absent (present in *P.scitulus*) and absence of marks in the groin or thighs (conspicuous dark spots in the groin that is continuous as marks on the posterior surfaces of the thighs in *P.scitulus*). *Pristimantistoftae* is a smaller species that is superficially similar to *P.similaris*. The new species can be distinghished by the absence of coloured marks or spots in the groin or other parts of its body (yellow spot in the groins of *P.toftae*), absence of labial bar (presence of a white labial bar in *P.toftae*).

*Pristimantissimilaris* is also similar to some species in the *Pristimantisconspicillatus* species Group, which includes *P.bipunctatus*, *P.skydmainos* and *P.iiap*. The parapatric *Pristimantisbipunctatus* (found in Calicanto at 1940 m. a.s.l. in the Piene Valley, Ayacucho Region), has dorsum and ventral skin shagreen and areolate, snout long, upper eyelids without tubercules similar to *P.similaris*, but the latter differs by having finger I slightly shorter than finger II (finger I and finger II about equal length in *P.bipunctatus*), discs on outer fingers truncated (broadly rounded in *P.bipunctatus*), scapular tubercules absent (present in *P.bipunctatus*) and by its smaller size (22.6–28.8 mm in males and 32.4–41.5 mm in females in *P.bipunctatus*). *P.similaris* can be distinguished from *P.skydmainos* by the absence of a mid-dorsal tubercle (present in *P.skydmainos*), absence of nuptial pads (present in *P.skydmainos*), finger I smaller than finger II (finger I longer than finger II in *P.skydmainos*), absence of spots or marks in the posterior surfaces of the thighs (minute cream flecks on the posterior surfaces of the thighs in *P.skydmainos*) and the absence of W-shaped marks (present in the scapular region in *P.skydmainos*). *Pristimantissimilaris* differs from *P.iiap* from the lowland Amazon of the Ucayali Region by lacking large granules on flanks (present in *P.iiap*), lacking granules on the upper eyelids (present in *P.iiap*) and by having finger I shorter than finger II (finger I and II about the same length in *P.iiap*).

Another species with some resemblance (mainly on the throat in ventral view) to the new species is *Pristimantistanyrhynchus*. *Pristimantissimilaris* can be distinguished from *P.tanyrhynchus* by the absence of nuptial pads in males (present in *P.tanyrhynchus*) and absence of tubercles on the heel (heel with a conical and large tubercle in *P.tanyrhynchus*).

##### Description of the holotype.

﻿Head longer than wide; head length 43% of SVL; head width 35% of SVL; cranial crests absent; snout subaccuminated in dorsal view and in lateral view (Fig. 4A, B, D); eye-nostril distance same as the eye diameter; nostrils slightly protuberant, directed dorsolaterally; canthus rostralis long, straight ﻿in lateral and in dorsal views; loreal region concave; upper eyelid without tubercles, width 90% of IOD (see photo in life Fig. 4); supratympanic fold short and narrow, extending from posterior margin of upper eyelid slightly curved to insertion of arm; tympanic membrane and annulus present; distinct conical postrictal tubercles present bilaterally. Choanae small, ovoid, not concealed by palatal shelf of maxilla; dentigerous processes of vomers absent; vocal slits present; tongue longer than short, oval, about a quarter times as long as wide, notched posteriorly, half of the tongue posteriorly free; one large vocal sac extending on to chest.

Skin on dorsum and flanks shagreen, continuous dorsolateral folds present extending from posterior level of tympanic area to level of hind limb insertion; skin on throat, chest and belly areolate; discoidal fold present; thoracic fold present.

Outer ulnar surface without tubercles; palmar tubercle bifid; thenar tubercle ovoid; subarticular tubercles well defined, most prominent on base of fingers, ovoid in ventral view, subconical in lateral view; supernumerary tubercles indistinct; fingers long and thin lacking lateral fringes, Finger I shorter than Finger II; tips of digits of fingers expanded, truncated, with circumferential grooves; nuptial pads absent (Fig. 5A).

Hind limbs long, slender, tibia length 58% of SVL; foot length 49% of SVL; dorsal surfaces of hind limbs tuberculate; inner surface of thighs smooth, posterior surfaces of thighs shagreen, ventral surfaces of thighs smooth; heels each with three small conical tubercles; outer surface of tarsus with one minute low tubercle; inner tarsal fold present; inner metatarsal tubercle ovoid, two times the size of round outer metatarsal tubercle; subarticular tubercles well defined, ovoid in ventral view, subconical in lateral view; few plantar supernumerary tubercles, about one quarter the size of subarticular tubercles; toes without lateral fringes; basal webbing absent; tips of digits expanded, truncated, less expanded than those on fingers, with circumferential grooves; relative length of toes: 1 < 2 < 3 < 5 < 4; Toe V slightly longer than Toe III (tip of digit of Toe III and Toe V not reaching distal subarticular tubercle on Toe IV; Fig. 5B).

##### ﻿Measurements (in mm) of the holotype.

﻿SVL 17.0; tibia length 9.9; foot length 8.4; head length 7.3; head width 5.9; eye diameter 2.3; inter orbital distance 1.9; upper eyelid width 1.7; internarial distance 2.0; eye-nostril distance 2.3; tympanum length 1.0; tympanum height 1.1; forearm length 4.3.

##### ﻿Colouration of the holotype in life

**(Fig. 4).** In life, dorsum dark brown with three conspicuous chevrons; flanks cream without tubercles, groin same pattern as flanks with orange-reddish colouration; anterior surfaces of thighs reddish-orange, posterior surfaces of thighs brown; venter cream to yellow with black conspicuous reticulations in the throat and black marks in the chest; iris dark copper-coloured with fine black vermiculations (Fig. 4).

##### ﻿Colouration of the holotype in preservative.

﻿The dorsal ground colouration is pale brown with three browner chevrons; narrow blackish canthal and supratympanic ﻿stripes; flanks pale brown with dark brown and cream flecks forming irregularly-shaped diagonal bars; groin and anterior surfaces of thighs brown with dark brown flecks; chest, belly and ventral surfaces of thighs pale cream, throat pale cream and grey-striped; palmar and plantar surfaces and fingers and toes dark brown; iris pale grey.

##### Variation.

All specimens have the same general appearance, with three chevrons on the dorsum. MUSM 41029 is completely yellow and lacks marks on the chest or throat. MUSM 41032 has two brown-yellowish longitudinal bars on the dorsolateral folds. MUSM 41341 is blackish-brown and the three chevrons are not very visible (Fig. 6). Some individuals (MUSM 41030–2, 41036–7) lack the dentigerous processes of vomers. Morphological measurements ranges and proportions of types are included in Tables 2, 3.

**Table 2.** Morphological measurements (mm) of nine paratype specimens of *Pristimantissimilaris* sp. nov. For abbreviations, see Materials and methods.

Character	MUSM 41028	MUSM 41029	MUSM 41031	MUSM 41032	MUSM 41033	MUSM 41034	MUSM 41035	MUSM 41036	MUSM 41037
Sex	Male	Male	Female	Female	Male	Male	Female	Female	Female
SVL	18.1	17.7	25.2	22.7	18.8	18.6	24.7	23.4	20.8
TL	10.3	10.4	14.8	13.5	10.5	10.8	14.0	14.1	12.1
FL	9.0	8.4	12.4	11.3	9.0	9.5	11.6	12.4	9.4
HL	7.2	7.2	9.6	8.9	7.3	7.9	9.8	9.2	8.9
HW	5.9	6.5	8.5	7.5	6.6	6.9	8.2	8.0	7.5
ED	1.9	2.4	2.8	2.5	2.3	2.3	2.9	2.6	2.4
TY	0.9	1.0	1.1	1.1	1.0	1.1	1.3	1.1	1.2
IOD	2.1	2.0	2.4	2.2	2.1	2.0	2.4	2.3	2.2
EW	1.8	1.7	2.3	1.9	1.8	1.8	2.3	2.2	2.1
IND	2.0	2.3	2.9	2.6	2.2	2.2	2.7	2.5	2.5
EN	2.3	2.3	3.1	2.8	2.5	2.6	3.0	2.7	2.8
F4	0.7	0.7	1.0	0.8	0.9	0.7	0.9	1.0	0.8

**Table 3.** ﻿Measurements (in mm) and proportions of adult male and female type specimens of *Pristimantissimilaris* sp. nov.; ranges followed by means and one standard deviation in parentheses. For abbreviations, see Materials and methods.

Character	Males (n = 5)	Females (n = 5)
SVL	17.0–18.6 (18.1 ± 0.7)	20.8–25.2 (23.4 ± 1.8)
TL	9.9–10.8 (10.4 ± 0.3)	12.1–14.8 (13.7 ± 1.0)
FL	8.4–9.5 (8.9 ± 0.5)	9.4–12.4 (11.4 ± 1.2)
HL	7.2–7.9 (7.4 ± 0.7)	8.9–9.8 (9.3 ± 0.4)
HW	5.9–6.9 (6.5 ± 1.0)	7.5–8.5 (7.9 ± 0.4)
ED	1.9–2.4 (2.2 ± 0.5)	2.4–2.9 (2.6 ± 0.2)
TY	1.0–1.1 (1.0 ± 0.1)	1.0–1.3 (1.2 ± 0.1)
IOD	1.9–2.1 (2.0 ± 0.1)	2.2–2.4 (2.3 ± 0.1)
EW	1.7–1.8 (1.75 ± 0.05)	1.9–2.3 (2.1 ± 0.2)
IND	2.0–2.3 (2.1 ± 0.1)	2.5–2.9 (2.6 ± 0.2)
EN	2.3–2.6 (2.4 ± 0.3)	2.8–3.1 (2.9 ± 0.2)
F4	0.7–0.9 (0.8 ± 0.1)	0.8–1.0 (0.9 ± 0.1)
TL/SVL	0.56–0.59	0.57–0.60
HL/SVL	0.39–0.43	0.38–0.43
EN/HL	0.32–0.34	0.30–0.33

##### Etymology.

The specific name corresponds to the Latin word “similar”. This refers to the similarity of the new species and its close phylogenetic relationship with *P.rhabdolaemus* and *P.pharangobates*.

##### Distribution and natural history.

The new species is only known from montane forests of Ayna and Anco in Departamento Ayacucho at elevations from 1200–2000 m a.s.l. in secondary forests (Figs 1, 3). This species was found only at night after 18:00 hours, usually perching on wet leaves 0.5–1.5 m above the ground. Males call rarely and their calls are overshadowed by other male species (*Pristimantislacrimosus* species Group) calling louder. The species is common and appears to tolerate some human disturbance, because it was found near abandoned farms, less frequented roads and in the sourroudings of abandoned houses. Syntopic species included candidate new species in the *Pristimantislacrimosus* species Group and candidate new species in the *Pristimantisplatydactylus* species Group, which were more abundant than the new species. Sympatric species included frogs and toads *Gastrothecapacchamama*, *Nympharguspluvialis*, *Boanapalaestes*, *Rhinellainca*, *Dendropsophusvraemi* and Hyalinobactrachiumaff.bergeri; lizards *Cercosauramanicata*, *Stenocercuscrassicaudatus* and *Potamitesmontanicola*; and snakes Dipsascf.peruana, *Leptodeiraannulata* and Epictiacf.peruviana.

## ﻿Discussion

We describe *Pristimantissimilaris*, a new species morphologically similar and phylogenetically related to *P.rhabdolaemus* and *P.pharangobates*. Despite their confusing taxonomic history (see Introduction), our phylogenetic analyses show that *P.rhabdolaemus* and *P.pharangobates* are distinct evolutionary lineages.

*Pristimantisrhabdolaemus* was described from mid-altitude montane forests restricted to Ayacucho and Cusco Departments (Duellman 1978). Although we visited the type locality of *P.rhabdolaemus* (Machente, Ayacucho Department) at 1650 m a.s.l., we could not find any specimens from this species. For that reason, we sequenced specimens of *P.rhabdolaemus* from Toccate, Anchihuay district in the Ayacucho Department (~ 38 km straight air line to Machente) because morphological analysis and comparison with the type series confirmed that these specimens corresponded to *P.rhabdolaemussensu stricto*. Padial et al. (2007) reported *P.rhabdolaemus* from Bolivia on the basis of 16S rRNA of two incorrectly assigned specimens (MNKA 6628 and MNCN 43036), but our concatenated phylogeny suggests that the Bolivian specimens belong to a different and probably undescribed species, *Pristimantis* sp. 3 (in purple, Fig. 2). Likewise, *P.pharangobates* should be restricted to Cusco Department until molecular data become available and support the presence of this species in Puno (south-eastern Peru) and Bolivia.

We also found another candidate species from Cosñipata and Alfamayo in Cusco, morphologically similar and phylogenetically related to *P.pharangobates* and *P.rhabdolaemus* (in blue, Fig. 2). Additional specimens and analyses are needed to assess the taxonomic status of these potential new species.

The taxonomy of other species of the *P.danae* species Group requires further work. For instance, specimens identified as *P.danae* or *P.reichlei* tend to form paraphyletic groups in phylogenies. We suggest that both species might benefit from future studies clarifying the phylogenetic relationships of their assigned populations. Such studies might include the use of genomic data for these species (including *P.toftae*) because the use of four genes (three nuclear) in this study was not sufficiently informative to infer with confidence phylogenetic relationships between the most inclusive clades.

Furthermore, our phylogenies include for the first time sequences of *P.scitulus* from Chungui, Ayacucho previously known only from two type specimens in Yuraccyacu, Ayacucho (at 2600 m a.s.l) and supports the assignment of this species in the *P.danae* species Group. We also included sequences for the first time of *P.iiap* (outgroup) and it is recovered in the *P.conspicillatus* species Group.

According to Swenson et al. (2012), who discussed the endemism of species (birds, mammals, plants and amphibians) in the eastern Andean slopes from the treeline (~ 3500 m a.s.l.) to the Amazon lowlands, most of the montane forests in the eastern Andean slopes of Peru and Bolivia (fig. 7 in Swenson et al. (2012)) are centres of endemism, specially in areas with little field evaluations due to social problems, such as montane forests in Ayacucho Department.

We would like to highlight the areas surroundings the type locality of *P.similaris* and closely-related species in south-eastern Peru. The Departments of Ayacucho and Cusco have biologically “irreplaceable areas” due to the configuration of the western Andes, the eastern Cordillera de Vilcabamba and the Apurimac River (Swenson et al. 2012). These geographical formations created a deep canyon along the Apurimac River at the border of the Departments of Ayacucho and Cusco (Lehr and Catenazzi 2008; Hazzi et al. 2018; Herrera-Alva et al. 2020), dissecting the Andean cordillera and providing mid-altitude isolated areas. The Apurimac Canyon is an important barrier for the dispersal of amphibians, such as high-altitude species of Terrarana: the Canyon splits the distribution of the genus *Phrynopus* to the northeast part of the Canyon from the distribution of *Bryophryne* southwest of the Canyon (Lehr and Catenazzi 2008, 2009, 2010). We believe that this pattern can be extended to mid-altitude montane forest frogs, such as species in the *P.danae* or *P.lacrimosus* species groups (pers. com. Ernesto Castillo-Urbina) or the distribution of mid-altitude toads, such as *Atelopusmoropukaqumir* (northwest of the Apurimac Canyon) and *A.erythropus* (southeast of the Canyon; Herrera-Alva et al. (2020)). Therefore, we hypothesise that the Apurimac Canyon could have promoted vicariant speciation of morphologically-similar *Pristimantis* in these montane forests. For instance, *P.similaris* occurs from 1200 to 2000 m a.s.l. on the northwest of the Apurimac Canyon in Ayacucho Department, while *Pristimantis* sp. ocurrs from 1200–2000 m a.s.l. in Cusco Department, southeast of the Apurimac Canyon. Nevertheless, the Apurimac Canyon might have not been a geographic barrier to other species, such as *P.rhabdolaemus* which has been found at both sides of the canyon. One population of this species has an altitude range from 2000–2900 m a.s.l. in Ayacucho (eastern part of the Apurimac River) and the other population ranges from 2000–2100 m a.s.l. in Cusco (western part of the Apurimac River) according to available specimens and sequences. The presence of *P.rhabdolaemus* on both sides of the Apurimac River will remain hypothetical until more specimens and tissues from Cusco Department become available and are tested against a hypothesis of two different species following an integrative approach.

We also provide information about infection by the fungus *Batrachochytriumdendrobatidis* (Bd). Chytridiomycosis, caused by the Bd fungus, has negatively affected amphibian communities in the montane forests of Central America and South America (Berger et al. 1998; Lips et al. 2008; Catenazzi et al. 2011; Catenazzi 2015). This pathogen has been associated with amphibian worldwide declines (Berger et al. 1998; Briggs et al. 2005; Lips et al. 2006; Catenazzi et al. 2010; Scheele et al. 2019). Catenazzi et al. (2011) reported a rapid decline in frog species richness and abundance from 1999 to 2008 in the upper Manu National Park (Cusco), which has communities and ecosystems similar to those found in our study area (Ayacucho). The high prevalence of 30% in *P.similaris* suggests that Bd could be threatening amphibians in the area and that Bd transmission (which is typically associated with aquatic species, given that the infective zoopores are aquatic) occurs in terrestrial frogs.

## Supplementary Material

XML Treatment for Pristimantis similaris

## Figures and Tables

**Figure A1.**
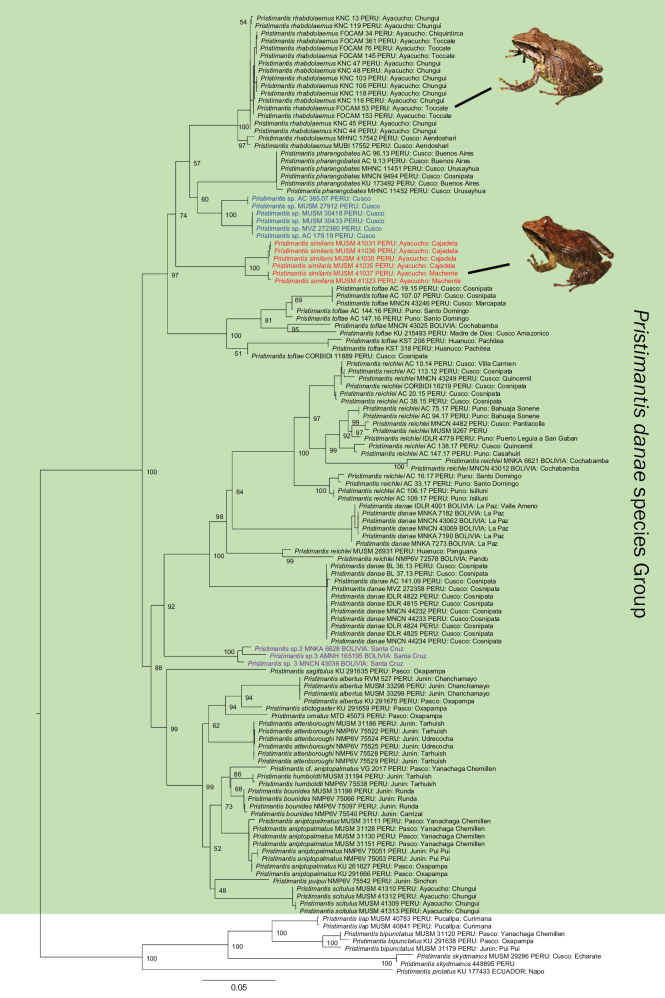
Maximum Likelihood tree non-collapsed of concatenated genes 16S rRNA, COI, RAG1 and TYR taken from GenBank and novel sequences. Numbers on nodes are bootstrap values (see Materials and Methods section for details). Green shadow corresponds to the ingroup. *Pristimantissimilaris* sp. nov. in red, *Pristimantis* sp. 3 from Bolivia in purple and *Pristimantis* sp. from Cusco in blue.

**Figure A2.**
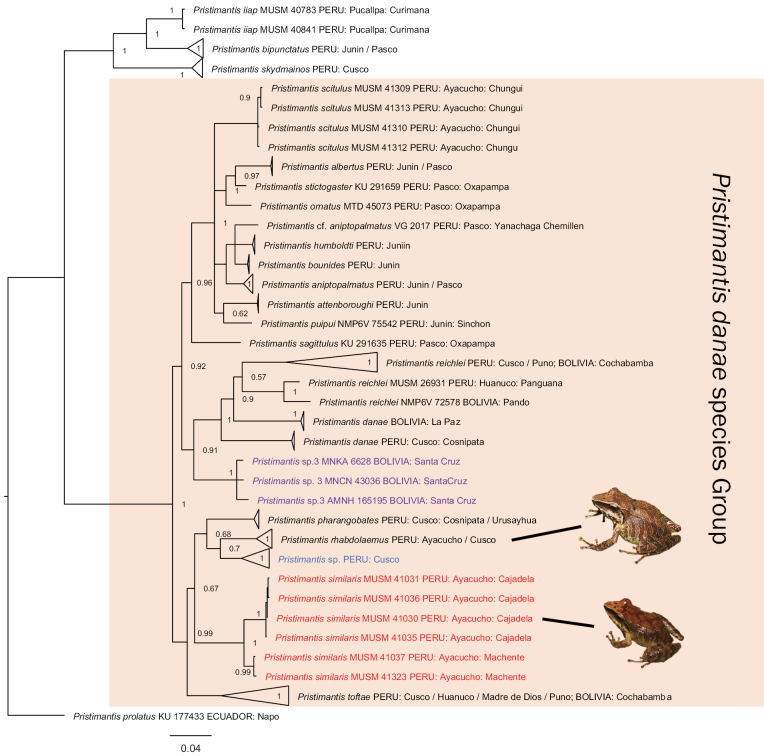
Bayesian Tree phylogeny collapsed of concatenated genes 16S rRNA, COI, RAG1 and TYR. Numbers on nodes are posterior probabilities (see Materials and Methods section for details). Orange shadow corresponds to the ingroup. *Pristimantissimilaris* sp. nov. in red, *Pristimantis* sp. 3 from Bolivia in purple and *Pristimantis* sp. from Cusco in blue.

## ﻿Additional information

### Author ORCIDs

Valia Herrera-Alva https://orcid.org/0000-0001-7858-8279

Alessandro Catenazzi https://orcid.org/0000-0002-3650-4783

Cesar Aguilar-Puntriano https://orcid.org/0000-0001-6372-7926

### Data availability

All of the data that support the findings of this study are available in the main text.

## ﻿Appendix 1

Specimens analysed from museums. See acronyms in Materials and methods. Underlined codes correspond to type material. Coordinates and altitude when available.

***Pristimantispharangobates***: AMNH 6099, 153089: Between Abra Accanaco and Pillahuata, Paucartambo, Cusco; KU 173236–173251: Buenos Aires, Cosñipata, Paucartambo, Cusco [-13.15; -71.5833; altitude: 2400 m a.s.l.]; MUBI 4203, 4205, 4209–10, 4212, 4217, 4220, 4222, 4224–4225, 4228, 4390–92, 4395–97, 4399–4400, 4560–61, 4563, 4610: Cosñipata, Paucartambo, Cusco [-13.1153; -715833; altitude: 2750 m a.s.l.]; MUBI 11451–52, 11374–87: Trocha Unión, Cosñipata, Paucartambo, Cusco [-13.1061; -71.5897; altitude: 2780 m a.s.l.]; MUSM 27910: Buenos Aires, Cosñipata, Paucartambo, Cusco; MUSM 32932–35, 32952–60: Paucartambo, Cusco.

***Pristimantisrhabdolaemus***: CORBIDI 10813, 10815–16: Chiquintirca, La Mar, Ayacucho [-13.0350; -73.6786; altitude: 2635 m a.s.l.]; LSUMZ 26150–51, 26153, 26156, 26182, 26251: Huanhuachayocc, Tambo to Apurimac Road [-12.75; -73.7833]; KU 175082–84: Huanhuachayocc, Tambo to Apurimac Road [-12.73; -73.7833]; MUBI 17541–42, 17552, 17555: Aendoshari Community, La Convención, Cusco [-12.8188; -73.4239; altitude: 2350 m a.s.l.]; MUSM 18505-08: Toccate, La Mar, Ayacucho [-12.9950; -73.6685; altitude: 2080 m a.s.l.]; 30206–08: Cielo Punku, Kimbiri, La Convención, Cusco [-12.8008; -73.5042; altitude: 2100 m a.s.l.]; KNC 44–45, 47–48, 106, 116, 118–20, 122, 130: Chaupichaca, Chungui, La Mar, Ayacucho [-13.1219; -73.5439; altitude: 2040–2345 m a.s.l.].

***Pristimantisscitulus***: KU 175085: Yuraccyacu, Tambo to Apurimac Road; LSUMZ 26097: Yuraccyacu, Tambo to Apurimac Road [-12.7833; -73.9]; MUSM 41309-10, 41312-13: Chaupichaca, Chungui, La Mar, Ayacucho [-13.12; -73.5228; altitude: 2270–2520 m a.s.l.].

***Pristimantis* sp.**: AMNH 153088, 153090: Between Accanaco and Pillahuata, Paucartambo, Cusco; KU 138877: 7 km WSW Santa Isabel, Cosñipata, Paucartambo, Cusco; MUBI 13317, 13323–24, 13333–34: San Pedro, Paucartambo, Cusco; MUSM 21035, 26269–70, 26276, 30418, 30433, 32934: Suecia, Paucartambo, Cusco. KU 175086–88: Huyro, Quillabamba, Echarate, Cusco; MUBI 13612, 13627, 13661: Mesa Pelada, Huayopata, La Convención, Cusco; MUSM 27911–12: Alfamayo, Huayopata, La Convención, Cusco.

***Pristimantis* sp. 4.**: LSUMZ 26147–48, 26154, 26158: Between Pataccocha and San Jose, Santa Rosa, Ayna, Ayacucho.

## ﻿Appendix 2

**Table A1.** Sequences used from GenBank and new sequences added in this paper.

N°	Species	Voucher	Group	Locality	Source	16 S	COI	RAG1	TYR
1	*Pristimantisalbertus*	KU 291675	Ingroup	Peru: Pasco, 0.9 km N, 2.1 km E Oxapampa	Hedges et. al (2008)	EU186695	–	–	–
2	*Pristimantisalbertus*	RvM 527	Ingroup	Peru: Junin, Provincia Chanchamayo, Puyu Sacha	Lehr and von May (2017)	KY594751	–	–	–
3	*Pristimantisalbertus*	MUSM 33299	Ingroup	Peru: Junin, Provincia Chanchamayo, Cerro San Pedro	Lehr and von May (2017)	KY594750	–	–	–
4	*Pristimantisalbertus*	MUSM 33298	Ingroup	Peru: Junin, Provincia Chanchamayo, Cerro San Pedro	Lehr and von May (2017)	KY594749	–	–	–
5	*Pristimantisaniptopalmatus*	KU 261627	Ingroup	Peru: Pasco, 2.9 km N, 5.5 km E Oxapampa	Hedges et. al (2008)	EF493390	–	–	–
6	*Pristimantisaniptopalmatus*	KU 291666	Ingroup	Peru: Pasco, 2.9 km N, 5.9 km E Oxapampa	Hedges et. al (2008)	EU186694	–	–	–
7	*Pristimantisaniptopalmatus*	NMP6V 75053	Ingroup	Peru: Junin, Pui Pui	Lehr et. al (2017a)	KY006087	–	–	–
8	*Pristimantisaniptopalmatus*	NMP6V 75051	Ingroup	Peru: Junin, Pui Pui	Lehr et. al (2017a)	KY006086	–	–	–
9	*Pristimantisaniptopalmatus*	MUSM 31151	Ingroup	Peru: Pasco, Yanachaga-Chemillen	Lehr et. al (2017a)	KY006085	–	–	–
10	*Pristimantisaniptopalmatus*	MUSM 31130	Ingroup	Peru: Pasco, Yanachaga-Chemillen	Lehr et. al (2017a)	KY006084	–	–	–
11	*Pristimantisaniptopalmatus*	MUSM 31128	Ingroup	Peru: Pasco, Yanachaga-Chemillen	Lehr et. al (2017a)	KY006083	–	–	–
12	*Pristimantisaniptopalmatus*	MUSM 31111	Ingroup	Peru: Pasco, Yanachaga-Chemillen	Lehr et. al (2017a)	KY006082	–	–	–
13	*Pristimantisattenboroughi*	NMP6V 75529	Ingroup	Peru: Junin, near trail from Tasta to Tarhuish, Polylepis forest patch	Lehr and von May (2017)	KY594757	KY962784	KY962764	–
14	*Pristimantisattenboroughi*	NMP6V 75528	Ingroup	Peru: Junin, near trail from Tasta to Tarhuish, Polylepis forest patch	Lehr and von May (2017)	KY594756	KY962783	KY962763	–
15	*Pristimantisattenboroughi*	NMP6V 75524	Ingroup	Peru: Junin, Upper part of Quebrada Tarhuish, ‘Laguna Udrecocha’	Lehr and von May (2017)	KY594754	KY962781	KY962761	–
16	*Pristimantisattenboroughi*	NMP6V 75522	Ingroup	Peru: Junin, Quebrada Tarhuish, left bank of Antuyo River	Lehr and von May (2017)	KY594753	KY962780	KY962760	–
17	*Pristimantisattenboroughi*	MUSM 31186	Ingroup	Peru: Junin, Quebrada Tarhuish, left bank of Antuyo River	Lehr and von May (2017)	KY594752	KY962779	KY962759	–
18	*Pristimantisattenboroughi*	NMP6V 75525	Ingroup	Peru: Junin, Upper part of Quebrada Tarhuish, ‘Laguna Udrecocha’	Lehr and von May (2017)	KY594755	KY962782	KY962762	–
19	*Pristimantisbounides*	NMP6V 75097	Ingroup	Peru: Junin, Quebrada Tasta, ‘’Runda’’	Lehr et. al (2017b)	KY962797	KY962790	KY962774	–
20	*Pristimantisbounides*	NMP6V 75066	Ingroup	“Peru: Junin, Sector Carrizal, Carrtera Satipo-Toldopampa, km 134”	Lehr et. al (2017b)	KY962796	–	KY962773	–
21	*Pristimantisbounides*	NMP6V 75540	Ingroup	“Peru: Junin, Sector Carrizal, Carrtera Satipo-Toldopampa, km 134”	Lehr et. al (2017b)	KY962795	–	KY962772	–
22	*Pristimantisbounides*	MUSM 31198	Ingroup	Peru: Junin, Quebrada Tasta, ‘’Runda’’	Lehr et. al (2017b)	KY962794	KY962789	KY962771	–
23	*Pristimantiscfaniptopalmatus*	VG-2017	Ingroup	Peru: Pasco, Yanachaga-Chemillen	Lehr et. al (2017a)	KY006088	–	–	–
24	*Pristimantisdanae*	MNCN 44234	Ingroup	Peru: Cusco, Union, Valle de Kosnipata	Padial and De la Riva (2009)	EU192270	–	–	–
25	*Pristimantisdanae*	IDLR 4001	Ingroup	Bolivia: La Paz: Santa Cruz de Valle Ameno	Padial and De la Riva (2009)	EU192260	–	–	–
26	*Pristimantisdanae*	MNK-A 7182	Ingroup	Bolivia: La Paz, Huairuro, senda San Jose - Apolo	Padial and De la Riva (2009)	EU192261	–	–	–
27	*Pristimantisdanae*	MNCN 43062	Ingroup	Bolivia: La Paz, Huairuro, senda San Jose - Apolo	Padial and De la Riva (2009)	EU192262	–	–	–
28	*Pristimantisdanae*	MNCN 43069	Ingroup	Bolivia: La Paz: Arroyo Huacataya. senda San José y Apolo	Padial and De la Riva (2009)	EU192263	–	–	–
29	*Pristimantisdanae*	MNK-A 7190	Ingroup	Bolivia: La Paz: Arroyo Huacataya. senda San José y Apolo	Padial and De la Riva (2009)	EU192264	–	–	–
30	*Pristimantisdanae*	MNK-A 7273	Ingroup	Bolivia: La Paz: Serranía Bella Vista	Padial and De la Riva (2009)	EU192265	–	–	–
31	*Pristimantisdanae*	IDLR 4815	Ingroup	Peru: Cusco: Unión, Valle de Kosñipata	Padial and De la Riva (2009)	EU192266	–	–	–
32	*Pristimantisdanae*	MNCN 44232	Ingroup	Peru: Cusco: Unión, Valle de Kosñipata	Padial and De la Riva (2009)	EU192267	–	–	–
33	*Pristimantisdanae*	MNCN 44233	Ingroup	Peru: Cusco: Unión, Valle de Kosñipata	Padial and De la Riva (2009)	EU192268	–	–	–
34	*Pristimantisdanae*	IDLR 4822	Ingroup	Peru: Cusco: Unión, Valle de Kosñipata	Padial and De la Riva (2009)	EU192269	–	–	–
35	*Pristimantisdanae*	IDLR 4824	Ingroup	Peru: Cusco: Unión, Valle de Kosñipata	Padial and De la Riva (2009)	EU192271	–	–	–
36	*Pristimantisdanae*	IDLR 4825	Ingroup	Peru: Cusco: Unión, Valle de Kosñipata	Padial and De la Riva (2009)	EU192272	–	–	–
37	*Pristimantisdanae*	MVZ 272358	Ingroup	Peru: Cusco: Valle de Kosñipata	von May et. al (2017)	KY652652	KY672984	KY672968	KY681073
38	*Pristimantisdanae*	AC 141.09	Ingroup	Peru: Cusco: Valle de Kosñipata	**This paper**	**OR469891**	–	–	**OR542804**
39	*Pristimantisdanae*	BL 37.13	Ingroup	Peru: Cusco: Valle de Kosñipata	**This paper**	**OR469893**	**OR478457**	**OR542831**	**OR542792**
40	*Pristimantisdanae*	BL 36.13	Ingroup	Peru: Cusco: Valle de Kosñipata	**This paper**	**OR469905**	**OR478456**	**OR542830**	**OR542791**
41	*Pristimantishumboldti*	NMP6V 75538	Ingroup	Peru: Junin, Quebrada Tarhuish, left bank of Antuyo River, Shiusha	Lehr et. al (2017b)	KY962799	KY962792	KY962776	–
42	*Pristimantishumboldti*	MUSM 31194	Ingroup	Peru: Junin, Quebrada Tarhuish, left bank of Antuyo River, Shiusha	Lehr et. al (2017b)	KY962798	KY962791	KY962775	–
43	*Pristimantisornatus*	MTD 45073	Ingroup	Perú: Pasco: Oxapampa: Chinche: Cerca de Aquimarca	Hedges et. al (2008)	EU186660	–	–	–
44	*Pristimantispharangobates*	KU 173492	Ingroup	Peru: Cusco: Buenos aires	Heinicke et. al (2007)	EF493706	–	–	–
45	*Pristimantispharangobates*	MNCN 9494	Ingroup	Peru: Cusco: Valle de Kosñipata	Padial and De la Riva (2009)	FJ438802	–	–	–
46	*Pristimantispharangobates*	MHNC 11451	Ingroup	Peru: Cusco: La Convención: Echarate: Urusayhua	**This paper**	**OR470757**	–	–	–
47	*Pristimantispharangobates*	MHNC 11452	Ingroup	Peru: Cusco: La Convención: Echarate: Urusayhua	**This paper**	**OR471343**	–	–	–
48	*Pristimantispharangobates*	AC 96.13	Ingroup	Peru: Cusco: Buenos Aires	**This paper**	**OR471423**	**OR478463**	–	**OR542798**
49	*Pristimantispharangobates*	AC 9.13	Ingroup	Peru: Cusco: Buenos Aires	**This paper**	–	**OR478451**	**OR542824**	**OR542787**
50	*Pristimantispuipui*	NMP6V 75542	Ingroup	Peru: Junin, Pui Pui Protected Forest, Laguna Sinchon	von May and Lehr (2017b)	KY962800	–	KY962777	–
51	*Pristimantisreichlei*	MUSM 9267	Ingroup	Perú	Heinicke et. al (2007)	EF493707	–	EF493436	EF493498
52	*Pristimantisreichlei*	MNCN 43012	Ingroup	Bolivia: Cochabamba: Los Guácharos	Padial and De la Riva (2009)	EU192287	–	–	–
53	*Pristimantisreichlei*	MNK-A 6621	Ingroup	Bolivia: Cochabamba: Los Guácharos	Padial and De la Riva (2009)	EU192286	–	–	–
54	*Pristimantisreichlei*	MNCN 43249	Ingroup	Peru: Cusco: 5 km from San Lorenzo hacia Quince Mil	Padial and De la Riva (2009)	EU192288	–	–	–
55	*Pristimantisreichlei*	IDLR 4779	Ingroup	Peru: Puno: Entre Puerto Leguia y San Gabán	Padial and De la Riva (2009)	EU192285	–	–	–
56	*Pristimantisreichlei*	MUSM 26931	Ingroup	Peru: Huanuco: Panguana	Pinto-Sanchez et. al (2012)	JN991461	JN991392	–	–
57	*Pristimantisreichlei*	CORBIDI 16219	Ingroup	Peru: Cusco: Valle de Kosñipata	von May et. al (2017)	KY652657	KY672989	KY672972	KY681078
58	*Pristimantisreichlei*	AC 38.15	Ingroup	Peru: Cusco: Valle de Kosñipata	**This paper**	**OR471610**	**OR478458**	**OR542832**	–
59	*Pristimantisreichlei*	AC 113.12	Ingroup	Peru: Cusco: Valle de Kosñipata	**This paper**	**OR471611**	**OR478467**	**OR542839**	**OR542801**
60	*Pristimantisreichlei*	AC 20.15	Ingroup	Peru: Cusco: Valle de Kosñipata	**This paper**	**OR471619**	**OR478454**	**OR542828**	–
61	*Pristimantisreichlei*	AC 138.17	Ingroup	Peru: Quispicanchi: Soqtapata: Quincemil	**This paper**	**OR471620**	**OR478469**	**OR542841**	**OR542803**
62	*Pristimantisreichlei*	AC 75.17	Ingroup	Peru: Puno: PN Bahuaja-Sonene: Punto 4	**This paper**	**OR471646**	**OR478460**	**OR542833**	–
63	*Pristimantisreichlei*	AC 94.17	Ingroup	Peru: Puno: PN Bahuaja-Sonene: Punto 4	**This paper**	**OR471650**	**OR478462**	**OR542835**	–
64	*Pristimantisreichlei*	AC 16.17	Ingroup	Peru: Puno, Inambari, Santo Domingo	**This paper**	**OR471653**	–	**OR542826**	–
65	*Pristimantisreichlei*	AC 33.17	Ingroup	Peru: Puno, Inambari, Santo Domingo	**This paper**	**OR475320**	–	**OR542829**	–
66	*Pristimantisreichlei*	AC 147.17	Ingroup	Peru: Puno: carretera San Gaban-Ollachea: Casahuiri	**This paper**	**OR471655**	**OR478472**	**OR542844**	**OR542807**
67	*Pristimantisreichlei*	AC 10.14	Ingroup	Peru: Cusco: Pilcopata: Villa Carmen	**This paper**	**OR471656**	**OR478452**	**OR542825**	–
68	*Pristimantisreichlei*	AC 106.17	Ingroup	Peru: Puno: Isilluni: Valle Limbani	**This paper**	**OR472333**	**OR478465**	**OR542837**	**OR542799**
69	*Pristimantisreichlei*	AC 109.17	Ingroup	Peru: Puno: Isilluni: Valle Limbani	**This paper**	**OR472388**	–	–	**OR542800**
70	*Pristimantisreichlei*	MNCN 4482	Ingroup	Peru: Cusco, Pantiacolla	Padial and De la Riva (2009)	EU712720	–	–	–
71	*Pristimantisreichlei*	NMP6V 72578	Ingroup	Bolivia: Pando, Bioceanica	Padial and De la Riva (2009)	EU712719	–	–	–
72	*Pristimantisrhabdolaemus*	FOCAM 34	Ingroup	Peru: Ayacucho: La Mar: Chiquintirca	**This paper**	**OR472495**	**OR478455**	**OR478455**	**OR542790**
73	*Pristimantisrhabdolaemus*	FOCAM 53	Ingroup	Peru: Ayacucho: La Mar: Toccate	**This paper**	**OR472500**	–	–	–
74	*Pristimantisrhabdolaemus*	FOCAM 76	Ingroup	Peru: Ayacucho: La Mar: Toccate	**This paper**	**OR472501**	**OR478461**	**OR542834**	**OR542797**
75	*Pristimantisrhabdolaemus*	FOCAM 145	Ingroup	Peru: Ayacucho: La Mar: Toccate	**This paper**	**OR472507**	–	–	–
76	*Pristimantisrhabdolaemus*	FOCAM 153	Ingroup	Peru: Ayacucho: La Mar: Toccate	**This paper**	**OR472508**	–	–	–
77	*Pristimantisrhabdolaemus*	FOCAM 361	Ingroup	Peru: Ayacucho: La Mar: Toccate	**This paper**	**OR472509**	–	**OR542849**	**OR542815**
78	*Pristimantisrhabdolaemus*	KNC 13	Ingroup	Peru: Ayacucho: Chungui: Chaupichaca	**This paper**	**OR472494**	–	–	**OR542788**
79	*Pristimantisrhabdolaemus*	KNC 47	Ingroup	Peru: Ayacucho: Chungui: Chaupichaca	**This paper**	**OR472498**	–	–	**OR542795**
80	*Pristimantisrhabdolaemus*	KNC 48	Ingroup	Peru: Ayacucho: Chungui: Chaupichaca	**This paper**	**OR472499**	–	–	**OR542796**
81	*Pristimantisrhabdolaemus*	KNC 103	Ingroup	Peru: Ayacucho: Chungui: Chaupichaca	**This paper**	**OR472502**	**OR478464**	**OR542836**	–
82	*Pristimantisrhabdolaemus*	KNC 106	Ingroup	Peru: Ayacucho: Chungui: Chaupichaca	**This paper**	**OR472503**	**OR478466**	**OR542838**	–
83	*Pristimantisrhabdolaemus*	KNC 118	Ingroup	Peru: Ayacucho: Chungui: Chaupichaca	**This paper**	**OR472505**	**OR478468**	**OR542840**	–
84	*Pristimantisrhabdolaemus*	KNC 119	Ingroup	Peru: Ayacucho: Chungui: Chaupichaca	**This paper**	**OR472506**	–	–	–
85	*Pristimantisrhabdolaemus*	KNC 116	Ingroup	Peru: Ayacucho: Chungui: Chaupichaca	**This paper**	**OR472504**	–	–	**OR542802**
86	*Pristimantisrhabdolaemus*	KNC 44	Ingroup	Peru: Ayacucho: Chungui: Chaupichaca	**This paper**	**OR472496**	–	–	**OR542793**
87	*Pristimantisrhabdolaemus*	KNC 45	Ingroup	Peru: Ayacucho: Chungui: Chaupichaca	**This paper**	**OR472497**	–	–	**OR542794**
88	*Pristimantisrhabdolaemus*	MHNC 17542	Ingroup	Peru: Cusco: La Convención: Echarate: Aendoshari	**This paper**	**OR472510**	**OR478479**	**OR542852**	–
89	*Pristimantisrhabdolaemus*	MUBI 17552	Ingroup	Peru: Cusco: La Convención: Echarate: Aendoshari	**This paper**	**OR472511**	–	–	**OR542819**
90	*Pristimantissagittulus*	KU 261635	Ingroup	Peru: Pasco, 0.9 km N, 2.1 km E Oxapampa	Heinicke et. al (2007)	EF493705	–	EF493439	EF493501
91	*Pristimantisscitulus*	MUSM 41310	Ingroup	Peru: Ayacucho: Chungui: Chaupichaca	**This paper**	**OR469744**	–	**OR542823**	–
92	*Pristimantisscitulus*	MUSM 41309	Ingroup	Peru: Ayacucho: Chungui: Chaupichaca	**This paper**	**OR469328**	–	–	–
93	*Pristimantisscitulus*	MUSM 41312	Ingroup	Peru: Ayacucho: Chungui: Chaupichaca	**This paper**	**OR469801**	–	–	–
94	*Pristimantisscitulus*	MUSM 41313	Ingroup	Peru: Ayacucho: Chungui: Chaupichaca	**This paper**	**OR469804**	–	–	–
95	***Pristimantissimilaris***	MUSM 41031	Ingroup	Peru: Ayacucho: La Mar: Cajadela	**This paper**	**OR478195**	**OR478473**	**OR542845**	**OR542808**
96	***Pristimantissimilaris***	MUSM 41030	Ingroup	Peru: Ayacucho: La Mar: Cajadela	**This paper**	**OR478198**	**OR478475**	**OR542847**	**OR542812**
97	***Pristimantissimilaris***	MUSM 41035	Ingroup	Peru: Ayacucho: La Mar: Cajadela	**This paper**	**OR478199**	**OR478476**	**OR542848**	**OR542813**
98	***Pristimantissimilaris***	MUSM 41036	Ingroup	Peru: Ayacucho: La Mar: Cajadela	**This paper**	**OR478200**	–	**OR542851**	**OR542817**
99	***Pristimantissimilaris***	MUSM 41037	Ingroup	Peru: Ayacucho: Ayna: Machente	**This paper**	**OR478196**	–	–	**OR542810**
100	***Pristimantissimilaris***	MUSM 41323	Ingroup	Peru: Ayacucho: Ayna: Machente	**This paper**	**OR478197**	–	–	**OR542811**
101	*Pristimantis* sp.	MVZ 272360	Ingroup	Peru: Cusco	von May et. al (2017)	KY652655	KY672987	KY681088	KY681076
102	*Pristimantis* sp.	MUSM 30433	Ingroup	Peru: Cusco	**This paper**	**OR478204**	–	**OR542855**	**OR542822**
103	*Pristimantis* sp.	MUSM 30418	Ingroup	Peru: Cusco	**This paper**	**OR478203**	–	**OR542854**	**OR542821**
104	*Pristimantis* sp.	AC 179.19	Ingroup	Peru: Cusco	**This paper**	–	–	**OR542846**	–
105	*Pristimantis* sp.	MUSM 27912	Ingroup	Peru: Cusco	**This paper**	**OR478202**	–	**OR542853**	**OR542820**
106	*Pristimantis* sp.	AC 365.07	Ingroup	Peru: Cusco	**This paper**	**OR478201**	**OR478478**	**OR542850**	**OR542816**
107	*Pristimantis sp3*	AMNH-A 165195	Ingroup	Bolivia: Santa Cruz: Caballero: Canton San José: Parque Nacional Amboro	Faivovich et. al (2005)	AY843586	–	–	–
108	*Pristimantis sp3*	MNK-A 6628	Ingroup	Bolivia: Santa Cruz: Serranía de la Siberia	Padial et. al (2007)	EU192258	–	–	–
109	*Pristimantis sp3*	MNCN 43036	Ingroup	Bolivia: Santa Cruz: La Yunga de Mairana	Padial et. al (2007)	EU192257	–	–	–
110	*Pristimantisstictogaster*	KU 291659	Ingroup	Peru: Pasco, 2.9 km N, 5.5 km E Oxapampa	Heinicke et. al (2007)	EF493704	–	EF493445	EF493506
111	*Pristimantistoftae*	KST 208	Ingroup	Peru: Huanuco, Puerto Inca, Panguana, Rio Yuyapichis (AKA Rio Llullapiches) near Rio Pachitea	Pinto-Sanchez et. al (2012)	JN991439	–	–	JN991566
112	*Pristimantistoftae*	KST 318	Ingroup	Peru: Huanuco, Puerto Inca, Panguana, Rio Yuyapichis (AKA Rio Llullapiches) near Rio Pachitea	**This paper**	**OR538542**	–	–	–
113	*Pristimantistoftae*	KU 215493	Ingroup	Peru: Madre de Dios: Cuzco Amazonico: 15 km E de Puerto Maldonado	Heinicke et. al (2007)	EF493353	–	–	–
114	*Pristimantistoftae*	MNCN 43025	Ingroup	Bolivia: Cochabamba: Los Guácharos	Padial and De la Riva (2009)	EU192293	–	–	–
115	*Pristimantistoftae*	MNCN 43246	Ingroup	Peru: Cusco: San Pedro, Valle de Marcapata	Padial and De la Riva (2009)	EU192294	–	–	–
116	*Pristimantistoftae*	AC 107.07	Ingroup	Peru: Cusco: Valle de Kosñipata	von May et. al (2017)	KY652659	KY672991	KY672974	KY681080
117	*Pristimantistoftae*	AC 19.15	Ingroup	Peru: Cusco: Valle de Kosñipata	**This paper**	**OR472575**	**OR478453**	**OR542827**	**OR542789**
118	*Pristimantistoftae*	CORBIDI 11889	Ingroup	Peru: Cusco: Valle de Kosñipata	**This paper**	–	–	–	**OR542818**
119	*Pristimantistoftae*	AC 144.16	Ingroup	Peru: Puno: Inambari: Santo Domingo	**This paper**	**OR472576**	**OR478470**	**OR542842**	**OR542805**
120	*Pristimantistoftae*	AC 147.16	Ingroup	Peru: Puno: Inambari: Santo Domingo	**This paper**	**OR472577**	**OR478471**	**OR542843**	**OR542806**
121	*Pristimantisbipunctatus*	MUSM 31120	Outgroup	Peru: Pasco, Yanachaga-Chemillen	Lehr et. al (2017a)	KY006089	–	–	–
122	*Pristimantisbipunctatus*	MUSM 31179	Outgroup	Peru: Junin, Pui Pui Protected Forest, Hito 3, Entrada del parque	Lehr and von May (2017)	KY594758	KY962785	KY962765	–
123	*Pristimantisbipunctatus*	KU 291638	Outgroup	Peru: Pasco, 0.7 km S, 4.5 km E Oxapampa	Heinicke et. al (2007)	EF493702	–	EF493430	EF493492
124	*Pristimantisiiap*	MUSM 40783	Outgroup	Peru: Ucayali: Pucallpa: Curimana	**This paper**	**OR470750**	**OR478474**	–	**OR542809**
125	*Pristimantisiiap*	MUSM 40841	Outgroup	Peru: Ucayali: Pucallpa: Curimana	**This paper**	**OR470749**	**OR478477**	–	**OR542814**
126	*Pristimantisprolatus*	KU 177433	Outgroup	Ecuador: Napo: Rio Salado	Hedges et. al (2008)	EU186701	–	–	–
127	*Pristimantisskydmainos*	MUSM 29286	Outgroup	Peru: Cusco: La Convención: Echarate	**This paper**	**OR469849**	–	–	–
128	*Pristimantisskydmainos*	448895	Outgroup	Peru	Heinicke et. al (2007)	EF493393	–	–	–

## Supplementary materials

Supplementary material 1 p-uncorrected distances of 591 pb including gaps of rRNA 16s gene Data type xls https://binary.pensoft.net/file/955211This dataset is made available under the Open Database License (http://opendatacommons.org/licenses/odbl/1.0/). The Open Database License (ODbL) is a license agreement intended to allow users to freely share, modify, and use this Dataset while maintaining this same freedom for others, provided that the original source and author(s) are credited.Valia Herrera-Alva, Alessandro Catenazzi, Cesar Aguilar-Puntriano
